# Relationship among motivation, vocabulary learning strategies and the breadth of vocabulary knowledge: A structural equation modeling study on second language learning

**DOI:** 10.1371/journal.pone.0356062

**Published:** 2026-09-22

**Authors:** Chunye Yang, Xuedong Yu, Hongshun Li

**Affiliations:** 1 School of International Communication, Wuhan Business University, Wuhan, Hubei, P. R. China; 2 Hubei Provincial Engineering Research Center of Racing Horse Detection and Application Transformation, Equine Science Research and Horse Doping Control Laboratory, Wuhan Business University, Wuhan, Hubei, P. R. China; 3 School of Foreign Languages, Zhongnan University of Economics and Law, Wuhan, Hubei, P. R. China; Bahir Dar University, ETHIOPIA

## Abstract

This study investigated Chinese second language learners’ learning motivation, second language learning (SLL) vocabulary learning strategies (VLSs), and their relationships with vocabulary knowledge (VK), especially with vocabulary size. Data were collected from 275 non-English major students at a university in Wuhan (230 valid responses, effective rate 83.63%), spanning from freshmen to seniors, predominantly composed of first-year and second-year students (90.9%), with limited representation from third- and fourth-year students across three semesters (2024 spring, 2024 autumn, and 2025 autumn). Questionnaires were used to gather demographic information, motivation, and VLSs for English learning. Vocabulary breadth was measured using the Vocabulary Size Test (VST). The VLSs questionnaire comprised three constructs: memory strategies, cognitive strategies, and metacognitive strategies. Structural equation modelling (SEM) was adopted to test the measurement model and the hypothesized structural model. Results demonstrated that (1) the sampled Chinese college students reported a moderate, slightly below average motivation level to study English vocabulary; (2) the sampled Chinese college students exhibited a preference for cognitive strategies in their English vocabulary learning; (3) a. EFL learners’ level of L2 motivation exerted a significant, positive direct effect on their use of VLSs (β = 0.796, p < 0.001); b. EFL learners’ level of L2 motivation exerted a moderate, positive direct effect on VK breadth (β = 0.294, p = 0.031); c. VLSs did not serve as a significant positive predictor of VK breadth (β = −0.097, p = 0.468); d. After controlling for VLSs, motivation had no significant indirect effect on VK breadth (Estimate = −2.692, 95% CI [−13.901, 6.863], p = 0.554), indicating the absence of mediation. These findings carry significant implications for both theory and practice. Theoretically, they enhance our understanding of the complex interplay among EFL learners’ motivation, VLSs, and VK. Practically, the insights can assist educators in integrating these constructs into instructional design to foster more efficient vocabulary acquisition and improve the effectiveness of college English teaching.

## 1. Introduction

Vocabulary learning is an important part of language learning [1,2] and an essential part of acquiring a language [2]. Vocabulary knowledge (VK) is affected by learners’ learning motivation and vocabulary learning strategies (VLSs). Certain types of VLSs may facilitate vocabulary acquisition [3]. In this regard, existing empirical studies had mainly focused on identifying the potential influential factors [4,5] and strategies [2,6] for promoting vocabulary learning, or the positive relationship between VLSs and VK [7,8]. The present study focuses on the latter aspect. To be more specific, this study focuses on the structural equation modelling (SEM) relationship among motivation, VLSs, and VK of the Chinese second language learning (SLL) learners. It aims to advance research on SLL, echoing the consistent call for continued yet renewed research in this domain.

Previous studies have investigated the impact of influential factors on language learning outcomes, including motivation [9], and different vocabulary learning strategies that successful and unsuccessful language learners adopt [10,11], online English studies [12], suggesting that motivation has a mediating function in promoting vocabulary proficiency, and that the way to deal with vocabulary contributed to the success/failure of foreign language learning. Further research also explored the roles of motivation or motivation beliefs in vocabulary learning, and the relationship between VLSs and VK. However, there are few studies on the complex relationship among motivation, VLSs, and VK in a Chinese SLL context. Therefore, this study aims to investigate the relationship among learning motivation, VLSs, and VK breadth.

Research questions of this study are as follows:

RQ1: What is the overall motivation level (ML) of participants for learning English as a second language, and how does ML differ across gender, academic major, and high school location (rural vs. urban)?

RQ2: Which VLSs are preferred by the sampled Chinese college students for English vocabulary learning, and how do VLSs preferences differ by gender, grade level, academic major, and high school location (rural vs. urban)?

RQ3: What are the relationships between: (a) motivation and VLSs, (b) motivation and the VK breadth, (c) VLSs and the VK breadth, (d) motivation, VLSs, and the VK breadth? It is hypothesized that H1: EFL learners’ motivation has a direct positive effect on their use of VLSs, H2: EFL learners’ motivation has a direct positive effect on their VK breadth, H3: EFL learners’ VLSs positively and directly predict their VK breadth, and H4: If VLSs plays a mediating role, motivation will exert an indirect effect on VK breadth.

## 2. Literature review

### 2.1. Motivation in second language learning

Motivation is the primary impetus initiating learning second language (L2) [13] and considered by many to be one of the main determining factors in success in developing a second or foreign language [14], as well as a key factor affecting SLL achievement [13,15] and SLL strategies that L2 learners adopt [13]. It has also been perceived as a pivotal element to affect the rate and success of second/foreign language, and provide driving force for L2 learners’ during the long and often tedious L2 learning process [13].

Motivation was frequently applied in research and education contexts, yet there was little agreement on the exact meaning of motivation in the literature. Gardner and Lambert [16], in 1972, classified second language learning motivation in two constructs: integrative motivation and instrumental motivation. The former reflected the identification to the culture of the target language while the latter was connected with the pragmatic benefits of the target language proficiency. Integrative and instrumental aspects were two emphasized parts of the prevalent motivation theory in second language learning, yet researchers are also dedicating broadening the theory. Therefore, studies on figuring out the other potential L2 learning motivation emerges. Deci and Ryan proposed and perfected self-determination theory in 1985 and 1995. This theory included two key conceptions: intrinsic motivation and extrinsic motivation. The extrinsic motivation covers three sub-constructs: external regulation, introjected regulation, and identified regulation. In 1992, Vallerand et al. developed intrinsic motivation into three constructs: intrinsic motivation-knowledge, intrinsic motivation-accomplishment, and intrinsic motivation-stimulation.

It was developed until the 20^th^ century that dynamic system theory (DST) was proposed by Zoltan Dörnyei [16]. DST includes three constructs: possible selves, ideal L2 self, and ought-to L2 self. The core idea of DST is the dynamic character of second language learning motivation. Based on the L2 Motivational Self System (L2MSS), the three components predict vocabulary learning strategies (VLSs) and vocabulary knowledge (VK) breadth through distinct mechanisms [17]. The Ideal L2 Self represents personal aspirations and intrinsic desires, generating promotion-focused motivation that drives learners to employ deep cognitive strategies (e.g., semantic mapping, contextual inferencing), thereby systematically expanding VK breadth [18,19]. The Ought-to L2 Self [20], rooted in external expectations and obligation avoidance, produces prevention-focused motivation that typically leads to superficial strategies such as rote repetition, exerting limited effects on VK breadth [5,20,21]. The L2 Learning Experience encompasses situational factors including classroom environment, teacher quality, and task enjoyment. A positive learning experience directly promotes engagement and strategy use, which in turn positively influences VK breadth [22]. As the research developed, from the 2010s, researchers have continued to apply Vygotsky’s sociocultural theory (originally proposed in the 1920s-1930s) to the study on second language learning.”

Motivation has a tight relationship with L2 learning strategies, L2 VK, and L2 instruction. Studies illustrated that motivation had a direct impact on the application of L2 learning strategies [14,23,24] and those who were unmotivated were insufficient to develop their L2 learning skills [14]. High motivation may promote VK development [25]. In China, Yining Zhang et al. surveyed the EFL students and found that intrinsic motivation had a positive and partial mediation effect on L2 VK while extrinsic motivation had a full mediation effect [8]. Jiajing Li, Chuang Wang [5], extracting data from 399 senior secondary school students in China, revealed the mediating role of motivation in the relationship between self-regulated learning capacity and vocabulary learning strategies. Meanwhile, L2 teaching instruction influenced EFL students’ learning motivation. For instance, Shujun Han [26] contended that blog-based writing instruction enhanced writing motivation of Chinese EFL learners. Abdullah Al Fraidan [27] surveyed sixty Saudi EFL students and stated that artificial intelligence (AI) tools and uncertain motivation (UM) strategies in teaching practice could effectively sustained student motivation. Lingjie Yuan and Xiaojuan Liu [28] demonstrated that application of AI tools affected the second language teaching practice had a beneficial effect on improving EFL students’ motivation. With quantitative analysis, Cuiping Song and Yanping Song [29] pointed out that AI-assisted instruction helped improve students’ academic writing skills and motivation.

Currently, studies on SLL motivation presented novel characteristics: (1) interdisciplinary integration: psychology, pedagogy, and other subjects are integrated with SLL motivation studies [30–32]; (2) dynamic studies on motivation [33,34]: studies on the dynamic process of motivation emerged and prevailed; (3) roles of the instructors in the instruction process [35,36]: more attention were given to the instructors and the instructors played a pivotal role in motivating and maintain the students’ learning motivation; (4) motivational performance in different cultural backgrounds: differences of motivational performance in various cultures aroused the researchers’ interests. Nigel Mantou Lou and Kimberly A. Noels [37] figured out that EFL students who can internalize the western and heritage culture would have more motivation to learn English and in turn predict the confidence to apply English; (5) influence of new technologies on motivation [38–40]: effect on motivation from digital learning.

Motivation studies in second language learning is of great importance in SLL and teaching. The relationship between motivation and SLL has also been fiercely discussed and deeply explored in China and in the other countries. However, there is a lack of empirical studies on the relationship among motivation, VLS, and VK breadth in China. Therefore, it is necessary and urgent to conduct such a study among Chinese college students on the EFL ML, and accordingly, the relationship among motivation, VLS, and VK.

### 2.2. VLSs of SLL

Mu-Hsuan Chou demonstrated as for “receptive and productive skills for effective comprehension and communication, vocabulary knowledge is an integral and indispensable part [41].” Vocabulary learning, to some extent, is a real key to SLL [42] since the mastery of a language skill relied heavily on how much VK the learners had possessed [43]. Norbert Schmitt [2] also pointed out that learning vocabulary is an essential part of mastering a second language. Vocabulary learning is about studies on the spelling, meaning, use of a single word or a multiword unit [44]. It is a practical tool to facilitate vocabulary acquisition [41] and essential for a second language competence [43]. Vocabulary learning is at the heart of language learning and vocabulary is the foundation to use and master a language. Students who had a large scale of vocabularies were considered relatively easier to enhance their language skills, thinking abilities, and communicating abilities [42] for VK is set up as a pivotal determiner for successful language communication [45].

VLSs have a close relationship with VK. According to the prior studies, VLSs have positive impact on VK, and using proper learning strategies directly affects learning a foreign language [46]. Orhan Kocaman and Gonca Kzlkaya Cumaolu [46] pointed out that “several studies have been consistent in their claims that language learning strategies and English proficiency are related”. Substantial researches were conducted to figure out what concrete VLSs could be utilized in VL process. In 1977, Schmitt, N [18]. firstly divided VLSs into two categories: discovery strategies and consolidation strategies. The former covers determination strategies and social strategies yet the latter includes memory, cognitive and meta-cognitive strategies. Orhan Kocaman and Gonca Kzlkaya Cumaolu [46] proposed that memory strategies are for storing and retrieving information; cognitive strategies for understanding and producing the language; and metacognitive strategies for planning and monitoring learning. Memory strategies incorporate the following specific VLSs: key words, executing manipulative mental processing, body gesture, repetition, roots and affixes, classification and grouping based on the themes, word property, synonyms and antonyms, mind mapping, memory palace, and interval review (e.g., review the new words according to the principle of The Ebbinghaus Forgetting Curve); cognitive strategies engage repetition, using mechanical means such as word lists and vocabulary notebooks, notetaking, practical application in speaking and writing, contexts, translation, and sentence building; metacognitive strategies include self-regulating one’s own vocabulary learning, monitoring learning process, self-assessment, and adjustment, and social strategies (e.g., learning or practicing vocabulary with peers).

To better acquire VK and enhance vocabulary learning efficiency, various VLSs were applied in the VK learning process. As for college students, selection and application of VLS is a hot topic [47], and scholars and researchers are contributing to identifying the optimal VLSs for there is no consensus on a universal learning strategy for most learners [46,47] to acquire VK effectively and quickly. Vocabulary learning strategy scales were developed in foreign language learning. Based on the Oxford’s (1990) sub-dimensions of Language Learning Strategies Scale, Orhan Kocaman and Gonca Kzlkaya Cumaolu [46] developed a vocabulary learning scale with 32 items and classified them into six dimensions: memory, cognitive, compensation, meta-cognitive, affective and social strategies. Adopting a quasi-experimental two-group pretest–post-test–delayed test design in the paper, Amjaad Mansour Alwadei & Mohammed Ali Mohsen investigated 41 Arab EFL learners and demonstrated that “infographics are effective in improving learners’ L2 vocabulary [43]”. In Iran, Ghasem Aghajanzadeh Kiasi and Abbas Pourhosein Gilakjani [48] tested the impact of definitional, sentential, and textual VLSs on Iranian EFL students. Mengsiying Li and Tai Wang [47], compared 12 common VLSs among East Asian college students in an empirical study, ranked the effective VLSs as digital flashcard strategy when used alone, paper flashcards, partner interaction game, and combination of the following three strategies—electronic diction, contextual guessing, and task-based reading. Fen Yang, I-Chun Lee, Christine Chifen Tseng and Siao-Cing Lai [49], surveyed 62 sixth graders in a primary school in central Taiwan, China, developed a self-regulated learning strategies, and demonstrated that digital game-based learning (DGBL) environment would encourage low-proficiency students to develop their memory, cognitive aspects of their self-regulated learning strategies to learn more new words. Ruofei Zhanga, Di Zoub and Gary Cheng also found that self-regulated digital game-based EFL vocabulary learning learners (SR-DGBVL) would experience significantly greater enhancement in VL [25] in the research conducted in a Chinese university. Keyi Zhou [50] et al., conducted a systematic review, found that these 40 of the retrieved 1221 journal articles from 2011 to 2023 all utilized cognitive strategies. They proposed that affective and social strategies should also be taken into consideration while currently cognitive strategies were dominant in vocabulary learning. other VLSs, such as repetition [51], content and language integrated learning (CLIL) [52], multi-media gloss in computer-assisted language learning (CALL) [53] were also tightly correlated with vocabulary gains.

Researches on EFL studies have been flourishing for a long time, but empirical studies investigating on the relationship between VLSs of SLL and VK among Chinese college students have been scarce. As for Chinese college students, how much they like to apply memory, cognitive and metacognitive strategies for EFL vocabulary learning, and how VLSs influence their VK deserve our attention.

### 2.3. VK breadth and its measurement of the EFL learners

As for first-and-second language researchers, vocabulary knowledge is of great significance in language competence [54]. Currently, how bilingual language speakers process the words, phrases, sentences in L2 learning aroused the cognitive researchers’ interests. The participants’ language proficiency, especially their vocabulary breadth caught researchers’ attention from psycholinguistic studies involving L2 learners [55]. Yet there was little consensus on how to measure word processing [55]. To objectively gauge L2 word aptitude, researchers were doing their utmost to develop tests, sub-tests or measures. To clearly illustrate the scattered pictures and the development of word aptitude of EFL learners’ learning, an overview of the typical English word aptitude tests in the recent five years’ studies was illustrated with a chronological sequence in Table 1.

**Table 1:** EFL learners’ word aptitude measurements since 2021.

Authors and articles	Topic	Word aptitude measurements
Raniya Abdullah Alsehibany [56] (2025)	integrating ChatGPT for VL and retention	vocabulary knowledge tests semi-structured interviews
Dongjing Han, David D Qian [57] (2024)	relationship between aural vocabulary knowledge and Word listening comprehension	Vocabulary Levels Test (VLT) Associates Test (WAT)
Bao Trang Thi Nguyen	role of vocabulary breadth headword	PDK assessment Long Quoc Nguyen [58] (2024) scoring in measuring productive Vocabulary derivative knowledge Levels Test
Burcu Güngör, Alev Önder	assessment tool for very young EFL Learners’	English young EFL Learners’ Picture (2023) [59]
Birger Schnoor et al. [60] (2023)	assessment of EFL development	C-test
Laura E.de Ruiter, Peizhi Eloi Puig-Mayenco et al. [61] (2023)	L2 global proficiency	VK LexTALE
Wen and Si Chen [62] (2022)	Testing Chinese children’s	Assessment of Chinese Children’s English vocabulary English Vocabulary test (ACCE-V)
Mostafa Janebi Enayat	contribution of VK to L2	Word Associates Test (WAT)
Ali Derakhshan [63] (2021)	speaking proficiency	Vocabulary Levels Test (VLT) Lex30, the speaking tasks of International English Language Testing System (IELTS)

From the table, it is clear that in the recent years’ researches, studies on VK was still in the center of second language learning since vocabulary acquisition was the cornerstone of SLL [56,64]. Diverse measurements of English vocabulary have been applied to test EFL learners’ VK and the role that VK has played in L2 learning. Vocabulary Levels Test (VLT), as the most representative vocabulary tests [65], is popular among scholars investigating EFL learners’ vocabulary size. Besides that, other researches on VK tests of SLL, such as Chinese (LexCHI [66]), Arabic (LexArabic [67]), Spanish (Lextale-Esp [68]), Portuguese (LextPt [69]), Italian (LexITA [70]), Chinese vocabulary proficiency test (CVPT) [71] were also conducted based on the methods LexTALE from or beyond Table 1. WAT, initially proposed by Sir Francis Galton in the late 19^th^ century and later developed by Read, J., was frequently adopted in EFL research to gauge L2 learners’ VK. Currently, as artificial intelligence (AI) develops, some digital ways were combined with vocabulary learning in EFL learning, including ChatGPT [56], gamified English vocabulary applications [72,73], digital storytelling (DST) [74], a mobile learning tool with a 5-step vocabulary learning (FSVL) strategy [75] and mobile devices [76], viewing L2 television [77], technology-mediated vocabulary teaching [78] and so on.

In China, as a second foreign language, English language was studied by majority of the non-English major college students. Enhancing their English language proficiency, to some extent, was the priority in foreign language studies. Since vocabulary plays a pivotal role in Chinese EFL learners’ L2 studies, psycholinguistics paid much attention to vocabulary studies. The current paper is also going to investigate their VK breadth with vocabulary size test (VST) in a Chinese college.

### 2.4. Relationship among motivation, VLS, and VK

Motivation and learning strategies were closely related [8] from a self-regulated learning perspective. Ryan and Deci pointed out that motivational factors were prerequisites for self-regulated learning in SLL [24]. Pintrich and De Groot demonstrated that cognitive and meta-cognitive learning strategies mediated the effect of motivation on L2 learning outcome [79]. Yining Zhang et al. [8], in the studies, showed that only a handful of research studied on the combined effect of motivation and learning strategies on SLL. Tsuda and Nakata [80] proposed that in EFL studies, cognitive strategies, metacognitive strategies, and motivation were very important components.

Since the inventory of VLS by Schmitt in 1997, several empirical studies have been conducted to test the relationship between VLS and VK, motivation and VK. Cong Wang, Sida Zhu, and Yanmei Dai [4], with the SEM method, found that VLSs, through the mediation of self-regulation, indirectly influenced the breadth of VK. They also illustrated that a positive correlation existed between VLSs and VK, and learners with a higher level of VK tend to utilize VLSs more effectively. Jiajing Li and Chuang Wang [5] revealed the mediating role of motivation between self- efficacy, motivation and vocabulary proficiency.

From the aforementioned literature, it could be confirmed that there were plenty of scholars interested in studying the relationship among motivation, VLSs and VK. Yet there is still a long way to go to figure out clearly the specific relationship among them since most of the current studies focused mainly on either motivation and VLSs, or VLSs and VK, or motivation and VK. Therefore, it is necessary to conduct such a study on the relationship among motivation, VLSs and VK in China, especially of the Chinese college students.

## 3. Materials and methods

### 3.1. Ethics statement

This study involved an anonymous questionnaire survey of students. The Academic Ethics and Integrity Committee of Wuhan Business University determined in 2024 that the study was exempt from full ethics review, as it involved no more than minimal risk and did not collect any personally identifiable information. A formal written confirmation of this exemption was issued on June 3, 2026, under approval number 2026-06-REC-01. The requirement for written informed consent was formally waived by the Committee because (1) the survey was completely anonymous, (2) participation involved no more than minimal risk to participants, and (3) non-participation did not affect academic standing. All participants were informed that their participation was voluntary, that they could withdraw at any time without consequence, and that their responses would remain anonymous. Neither participation nor non-participation had any influence on course grades or academic evaluation.

### 3.2. Data acquisition

This exploratory study was operated in Wuhan, Hubei Province in inland China with an online questionnaire on the platform *Wen Juanxing*, which is a popular platform to conduct questionnaire. Website of this research questionnaire is: https://ks.wjx.com/vm/mBhPbDD.aspx#, yet the designer manually closed the response function, rendering the webpage inaccessible now.

The questionnaire consists of four parts. Part One is the demographic information of the participants, including gender, grade, major, English score of the College Entrance Exam, and location of the high schools. Part Two is the questionnaire on motivation, followed by a questionnaire on vocabulary learning strategies (memory, cognitive, and metacognitive), and questionnaires on vocabulary size test as Part Three and Part Four.

The motivation test was adapted from the L2MSS theory which was proposed by Zoltan Dörnyei in 2005. This system includes Ideal L2 self (IS), Ought-to L2 self (OS), and L2 learning experience (LLE), containing 15 items (e.g., I imagine myself having fluent conversations with overseas friends in English.), 5 items (e.g., Learning English is important because the people around me want me to study English.) and 28 items (e.g., Learning a L2 is enjoyable and fun for me) in the questionnaire respectively. All items used a five-point Likert-type scale, ranging from “1 = Never” to “5 = Usually” (1 = never, 2 = seldom, 3 = sometimes, 4 = often, 5 = usually). A five-point Likert-type scale was found appropriate to do such data analysis, such as t-test, linear regression, factor analysis, structural equation modeling, and prediction model construction approaches [81].

Questionnaire on vocabulary learning strategies includes three constructs: memory strategies, cognitive strategies, and metacognitive strategies, each containing 13 items (e.g., I orally repeat a new word, its related phrase or the example sentence.), 12 items (e.g., I try to use new words as much as possible in my speaking), and 7 items (e.g., In using new words, I guess their collocations, that is, words frequently co-occurring with them.). As for cognitive strategies, it covers 5 items for guessing, 4 for dictnote, and 3 for attention. All items used a five-point Likert-type scale, ranging from “1 = Never” to “5 = Usually” (1 = never, 2 = seldom, 3 = sometimes, 4 = often, 5 = usually).

Questionnaire on VK is to test the participants’ vocabulary size (VK breadth). It was adopted from the Vocabulary Size Test (VST) of Nation, I. S. P., & Beglar, D.. The website is: https://www.wgtn.ac.nz/lals/resources/paul-nations-resources/vocabulary-tests/the-vocabulary-size-test/fourteen-thousand-vocabulary-size-test-answers.pdf. As is described in the introduction to VST, the 14,000-word version is designed to measure total receptive vocabulary size, including levels beyond learners’ current knowledge. Research indicates that successful non-native undergraduate students typically have 5,000–6,000 word families, while PhD students reach about 9,000. Achieving 98% text coverage for reading novels and newspapers requires 8,000–9,000 word families. By comparison, average 13-year-old native speakers know 10,000–11,000 word families. Even lower-proficiency learners should complete all levels, as they may recognize some low-frequency words through loanwords or personal interests. However, unmotivated learners who guess randomly may receive substantially underestimated scores.

*Circle the letter a-d with the closest meaning to the key word in the question.*

*E.g., SEE: They* ***saw*** *it.*

*a. cut b. waited for c. looked at d. started.*

The VST measures total vocabulary size (i.e., how many words someone knows). The VST includes 14,000-word families based on particular frequency levels (e.g., 1000, 2000, 3000, 4000…and 14000) to complete with only one correct answer for each item. The participants’ vocabulary size equals to 100 * number of the questions that the participants got right.

The VST was administered in a single session via an online platform without real-time proctoring. To mitigate extraneous time pressure, the test was self-paced and no time limit was imposed. Participants were explicitly instructed to refrain from consulting dictionaries, translation applications, or any external resources and were required to complete the test independently. To safeguard research integrity, participants were informed that honest responding was expected. Cases exhibiting implausible response patterns, such as identical answers across all items, were excluded from subsequent data analyses.

### 3.3. Demographic information of the participants and their English language proficiency

A total of 275 responses (N = 275) from the participants who were non-English major undergraduates were collected, yet those obviously incorrect or incomplete answers were deleted, leaving 230 valid (effective rate 83.64%) answers for the following research and data analysis. Participants include: 155 freshmen (67.39%), 54 sophomores (23.48%), 19 juniors (8.26%), and 2 seniors (0.87%) of a university in Wuhan City, Hubei Province, China. 73 (31.74%) of the participants were male and 157 (68.26%) of them were female; 103 (44.78%) of them were majoring in Humanities (except English), and 127 (55.22%) of them were Science and Engineering. As for the location of the participants’ high school, 118 (51.3%) was in the urban areas, while 112 (48.7%) were in the rural areas (see Table 2).

**Table 2:** Demographic information and participants composition (N = 230).

Variable	Category	Frequency	Percentage (%)
Gender	Male	73	31.74
	Female	157	68.26
Grade	Freshman	155	67.39
	Sophomore	54	23.48
	Junior	19	8.26
	Senior	2	0.87
High School Location	Rural Area	112	48.7
	Urban Area	118	51.3
University	Wuhan Business University	224	97.39
	Others (scattered responses)	6	2.61
Major Type	Humanities (excluding English majors)	103	44.78
	Science and Engineering	127	55.22

Cronbach’s α was tested with SPSS 26.0. Internal consistency for each factor and the engagement scale was assessed using Cronbach’s α. Values ≥ 0.70 were considered acceptable, ≥ 0.80 good, and ≥ 0.90 excellent. Dimension Cronbach’s α, Variable Cronbach’s α, and Scale Cronbach’s α were all above 0.8, showing a good consistency and reliability of the scale. Cronbach’s α for scale: Cronbach’s α (ML) was 0.961, Cronbach’s α (VLSs) was 0.968. Cronbach’s α for IS was 0.958, OS 0.832, LLE 0.938, and VLSs 0.968 respectively (see Table 3).

**Table 3:** Reliability test (N = 230).

Scale	Variable	Dimension	Items	Dimension Cronbach’s α	Variable Cronbach’s α	Scale Cronbach’s α
Motivation	Ideal L2 Self	Ideal L2 Self	15	0.958	0.958	0.961
	Ought-to L2 Self	Ought-to L2 Self	6	0.832	0.832	
	Learning Experience	LLE-Positive Emotion	6	0.923	0.938	
		LLE-Negative Emotion	5	0.825		
		LLE-Engagement	3	0.861		
		LLE-Relationship	4	0.839		
		LLE-Meaning	4	0.929		
		LLE-Accomplishment	6	0.898		
VLSs	VLSs	VLS-Memory	13	0.941	0.968	0.968
		VLS-Cognitive	9	0.909		
		VLS-Metacognitive	10	0.934		

KMO and Bartlett’s test were conducted to test the reliability of the questionnaire. From Table 4, it could be seen that KMO = 0.925 > 0.6, Bartlett’s test of sphericity was significant (p < 0.001), showing a good reliability of the questionnaire.

**Table 4:** Validity test (KMO and Bartlett test, N = 230).

KMO		0.925
Bartlett’s Test of Sphericity	Approx. Chi-Square	17052.562
	df	3240
	Sig. (p-value)	0

**Fig 1:**
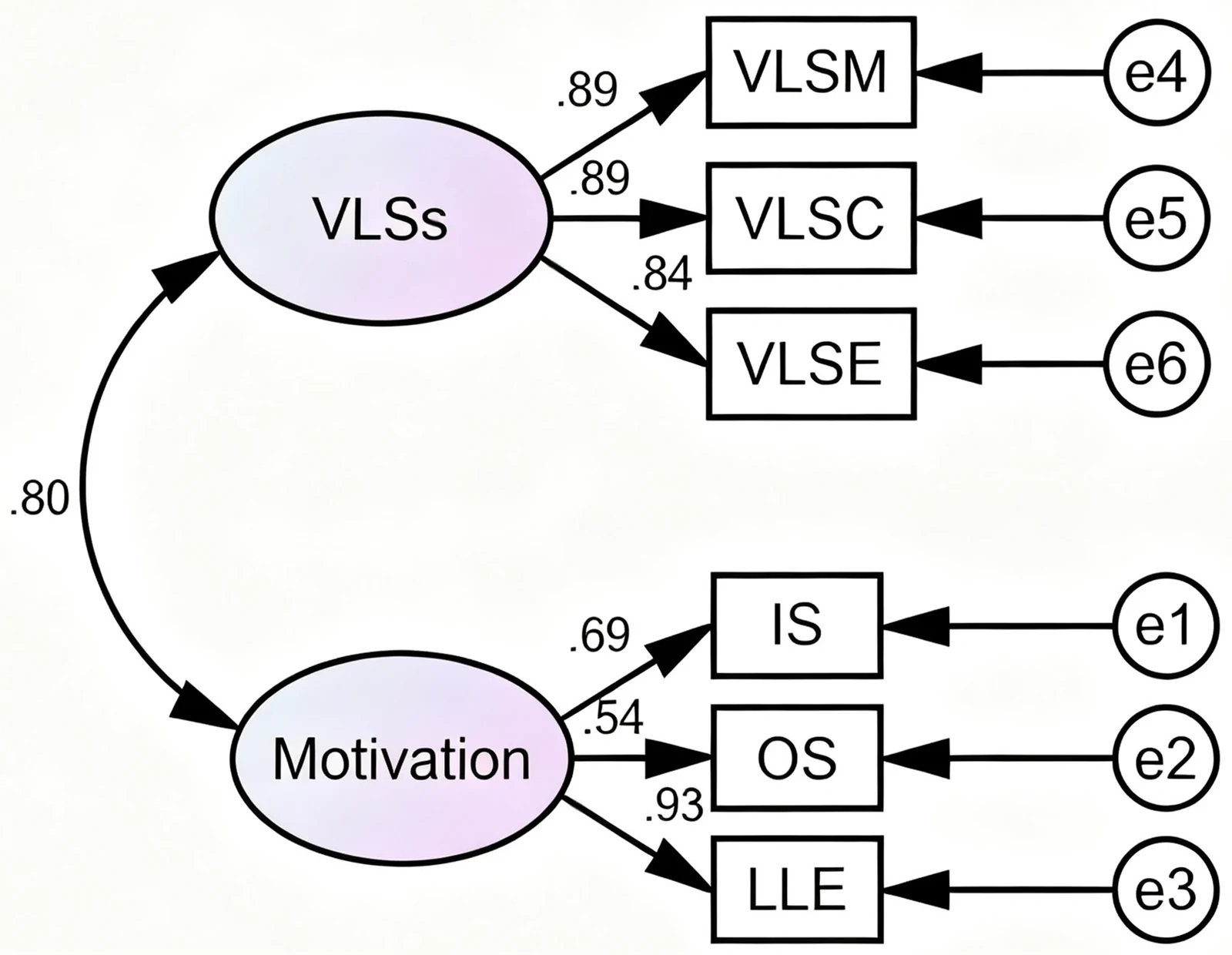
Standardized coefficients for the overall CFA Model.

A confirmatory factor analysis (CFA) test was also conducted (see Fig 1) to assess the convergent validity and composite reliability of the overall model. Data in Table 5 demonstrated that the overall reliability and validity levels of the two latent variables, Motivation and VLSs, both reached acceptable standards, CR (Motivation)=0.774 > 0.5, CR (VLSs)=0.905. Standardized factor loadings of IS = 0.692, OS = 0.539, LLE = 0.93, VLSM = 0.889, VLSC = 0.886, VLSE = 0.841. AVE (motivation)=0.544 > 0.5, AVE (VLSs)=0.761 > 0.5, exhibiting a satisfactory convergent validity.

**Table 5:** Overall reliability and validity test of the model.

Variable	Dimension	Estimate	S.E.	CR	AVE
Motivation	IS	0.692		0.774	0.544
	OS	0.539	0.098		
	LLE	0.93	0.083		
VLSs	VLSM	0.889		0.905	0.761
	VLSC	0.886	0.053		
	VLSE	0.841	0.058		

The overall discriminant validity of the model was tested by using the Fornell–Larcker criterion. As shown in Table 6, the square root of the AVE for motivation is 0.738, which is greater than its correlation with VLSs (0.629); the square root of the AVE for VLSs is 0.872, which is also greater than its correlation with Motivation (0.629). This indicates that each latent variable is more strongly associated with its own indicators than with the other latent variable.

**Table 6:** Discriminant validity of the overall model.

	Motivation	VLSs
Motivation	0.738	
VLSs	0.629	0.872
The diagonal values are the square roots of AVE.		

Overall, the square roots of the AVE for both latent variables (Motivation and VLSs) are higher than the correlations between the variables, meeting the Fornell–Larcker criterion. This suggests that the overall model has good discriminant validity, and the latent variables demonstrate adequate distinctiveness, reflecting different theoretical constructs.

It was a pity that two more answers (526 points, 150 points) were deleted, leaving only 228 (230 students in total,) valid answers to the question “English grade of the College Entrance Exam (150 points in total)” were collected. Luckily, it had only little influence on the data analysis for the approximate error was within a reasonable range. Therefore, N = 228 was used to test *Z* scores of the questionnaire to test the participates’ language proficiency. Of the 228 valid answers, the highest score was 133 points, yet the lowest was 20 points. The average point of the valid answers was 102.23 points. 28 (12.28%) of the participants’ score ranged from 120 to 150; 158 (69.298%) of the participants’ score ranged from 90 to 119. However, 42 (18.42%) of the participants could not pass the exam, which means that the score was lower than 90 (90 not included here).

Freshmen and sophomores (85.46%) were of the majority while juniors and seniors were also covered, because freshmen and sophomores were still having the face-to-face instruction in the classroom with the instructors, yet the juniors and senior had no more offline college English classes. Although the juniors and senior had no more face-to-face English classes in the classroom, they still need to study English so as to be prepared for the tests or further studies, such as CET4, CET6, GRE, GMAT, overseas studies, or postgraduate qualifying examination. Participants have been studying English since junior high school. They had little or no exposure to the English language outside the classroom. Therefore, they could be regarded as EFL learners.

Prior studies have been conducted in universities from the eastern districts such as Beijing, Southern China, relatively-less-developed districts such as Shanxi, Chongqing and Henan Province. Therefore, universities in this study were chosen from mid-China where it lacks research of this kind.

Demographic information regarding grade, discipline, and location of their high schools was collected (see Table 2). Participants were also asked to report their English scores of the College Entrance Exam (Gaokao English score, total: 150 points), yet only 228 (82.91%) of the 275 participants responded in the questionnaire, whose scores ranged from 20−133 points. To meet the demand of the multi-group analysis in SEM and analysis of between-group variance, participants were divided into three proficiency groups, i.e., high, mid, and low based on *z*-scores, i.e., *z* > 1 for high, −1 < *z* ≤ 1 for mid, and *z* ≤ −1 for low (see Table 3).


$$\begin{array}{c} \text{z }=(\text{X }-\mu )/\sigma \end{array}$$

*z*-scores refers to standard score; X refers to the original English score in the College Entrance Exam of the participants; μ refers to the average score; σ refers to standard deviation (SD). From the data, it is calculated that μ = 102.2316, σ (SD)=16.8726. Through data analysis, scores of the Gaokao English score: 120–133 points, 86–119 points, 20–85 points demonstrate high, mid, and low English language proficiency respectively. Data showed that 28 (12.3%) of the 228 participants were of high proficiency, 178 (78.1%) were of mid proficiency, and 22 (9.6%) were of low proficiency. In the light of the results, 90.4% of the responded participants (N = 228) were of high or mid proficiency (see Table 7), which also suggested that the VST was suitable for the EFL learning participants in a Chinese context.

**Table 7:** English language proficiency of the surveyed participants (N = 228).

Proficiency (N = 228)			English scores of the College Entrance Exam (N = 228,150 points in total)
High	Mid	Low	Lowest: 20 points Highest: 133points Average: 102.23points μ = 102.2316, σ (SD)=16.8726
28 (12.3%)	178 (78.1%)	22 (9.6%)	
Original score	Original score	Original score	
120-133 points	86-119 points	20-85 points	

## 4. Results

**RQ1: What is the overall motivation level (ML) of participants for learning English as a second language, and how does ML differ across gender, academic major, and high school location (rural vs. urban)?**

Data in Table 8 revealed that mean score (M) and standard deviation (SD) of the participants’ ML in the current study was M ± SD = 2.891 ± 1.075. According to the theory, the ML score of the ideal L2 self was M ± SD = 2.73 ± 1.105; ML score of the ought-to L2 self was M ± SD = 2.774 ± 1.133; ML score of the L2 learning experience was M ± SD = 3.169 ± 0.988. As for a five-point Likert-scale, this was of a moderate, slightly below average motivation level on the whole.

**Table 8:** Indicators of Chinese EFL learners’ ML.

Indicators						
Items	N	Minimum	Maximum	M	SD	Median
IS1	230	1	5	2.496	1.06	2
IS2	230	1	5	2.813	1.123	3
IS3	230	1	4	1.983	0.901	2
IS4	230	1	5	2.678	1.137	3
IS5	230	1	5	2.504	1.089	2
IS6	230	1	5	2.861	1.073	3
IS7	230	1	5	2.435	1.138	2
IS8	230	1	5	2.652	1.168	3
IS9	230	1	5	2.913	1.07	3
IS10	230	1	5	3.304	1.111	3
IS11	230	1	5	2.609	1.115	2
IS12	230	1	5	3.27	1.217	3
IS13	230	1	5	3.087	1.11	3
IS14	230	1	5	2.726	1.136	3
IS15	230	1	5	2.617	1.126	2
Average				2.73	1.105	
OS1	230	1	5	3.017	1.163	3
OS2	230	1	5	2.883	1.182	3
OS3	230	1	5	2.809	1.155	3
OS4	230	1	5	2.348	1.007	2
OS5	230	1	5	2.8	1.157	3
OS6	230	1	5	2.787	1.134	3
Average				2.774	1.133	
LLEP1	230	1	5	2.943	1.066	3
LLEP2	230	1	5	3.052	1.048	3
LLEP3	230	1	5	3.417	1.019	3
LLEP4	230	1	5	2.965	1.036	3
LLEP5	230	1	5	3.196	1.024	3
LLEP6	230	1	5	3.287	1.051	3
LLEN1	230	1	5	2.896	0.914	3
LLEN2	230	1	5	2.7	0.916	3
LLEN3	230	1	5	2.713	0.991	3
LLEN4	230	1	5	3.026	1.019	3
LLEN5	230	1	5	2.891	0.882	3
LLEE1	230	1	5	3.23	0.932	3
LLEE2	230	1	5	3.291	0.919	3
LLEE3	230	1	5	3.148	0.894	3
LLER1	230	1	5	3.252	1.01	3
LLER2	230	1	5	3.748	1.044	4
LLER3	230	1	5	3.17	1.05	3
LLER4	230	1	5	3.2	1.067	3
LLEM1	230	1	5	3.591	1.04	4
LLEM2	230	1	5	3.643	1.038	4
LLEM3	230	1	5	3.648	1.02	3.5
LLEM4	230	1	5	3.548	1.088	3
LLEA1	230	1	5	3.013	0.978	3
LLEA2	230	1	5	2.874	0.965	3
LLEA3	230	1	5	2.983	0.866	3
LLEA4	230	1	5	3.157	0.883	3
LLEA5	230	1	5	2.917	0.933	3
LLEA6	230	1	5	3.226	0.976	3
Average				3.169	0.988	
Overall M ± SD = 2.891 ± 1.075						

As for English learners with different genders, majors, high school locations (urban/ rural areas), data also showed a moderate, slightly below average motivation level, M ± SD = 2.97 ± 1.11, and M ± SD = 2.81 ± 1.03, respectively. The IS, OS, LLE score of the male respondents were M ± SD = 2.44 ± 1.13, 2.58 ± 1.20, 2.88 ± 1.04, while that of the female respondents were M ± SD = 2.82 ± 1.07, 2.86 ± 1.09, 3.27 ± 0.94. the overall average ML score of the male respondents and the female ones were M ± SD = 2.63 ± 1.12, 2.98 ± 1.03 respectively. The ML mean scores of freshmen, sophomores, juniors, and seniors were M ± SD = 2.94 ± 1.06, 2.82 ± 1.08, 2.63 ± 1.08, 2.73 ± 0.96, respectively. The ML mean scores of different majors were 2.89 ± 1.01 for Humanities (English excluded) and M ± SD = 2.88 ± 1.13 for Science and Engineering. The ML mean scores of the high school locations were M ± SD = 2.97 ± 1.11 for urban areas and 2.81 ± 1.03 for rural areas. This meant that most of the sampled Chinese college students had a moderate, slightly below average motivation level to study English as a second language (see Table 9).

**Table 9:** ML of the EFL learners with different genders, grades, majors, and high school locations.

Variables		ML(M ± SD)			
		IS	OS	LLE	Average
Genders	Male	2.44 ± 1.13	2.58 ± 1.20	2.88 ± 1.04	2.63 ± 1.12
	Female	2.82 ± 1.07	2.86 ± 1.09	3.27 ± 0.94	2.98 ± 1.03
Grades	Freshmen	2.79 ± 1.09	2.79 ± 1.13	3.25 ± 0.97	2.94 ± 1.06
	Sophomore	2.69 ± 1.10	2.77 ± 1.15	3.00 ± 0.99	2.82 ± 1.08
	Junior	2.27 ± 1.09	2.66 ± 1.14	2.97 ± 1.01	2.63 ± 1.08
	Senior	2.43 ± 0.86	2.75 ± 1.06	3.02 ± 0.96	2.73 ± 0.96
Majors	Humanities	2.78 ± 1.05	2.72 ± 1.01	3.16 ± 0.96	2.89 ± 1.01
	Science and engineering	2.66 ± 1.15	2.82 ± 1.23	3.16 ± 1.00	2.88 ± 1.13
Locations	Urban	2.83 ± 1.15	2.83 ± 1.17	3.24 ± 1.01	2.97 ± 1.11
	Rural	2.62 ± 1.05	2.72 ± 1.09	3.09 ± 0.96	2.81 ± 1.03

The data demonstrated that female students, compared with male students, had a higher ML to study EFL. As for students of different grades, freshmen ranked the first, followed by sophomore, and seniors, and juniors. It is appreciated that freshmen, after the College Entrance Exam, entering to a new campus, they can still have the passion to study new things. As for sophomores, they need to prepare for the CET4, thus they are faced up with high pressure. Seniors, who will soon graduate from the campus, surprisingly showed a relatively high ML for some of them want to have further studies or doing abroad.

**RQ2: Which VLSs are preferred by the sampled Chinese college students for English vocabulary learning, and how do VLSs preferences differ by gender, grade level, academic major, and high school location (rural vs. urban)?**

Data in Table 10 demonstrated that mean scores and SD was M ± SD = 3.02 ± 0.984, indicating that the sampled Chinese college students would use VLSs in English vocabulary learning. M ± SD of memory strategies, cognitive strategies, and meta-cognitive strategies in learning English were M ± SD = 2.98 ± 1, 3.191 ± 0.967, 2.889 ± 0.986, respectively, illustrating that cognitive strategies were the preferred L2 VLS, and meta-cognitive strategies were the least frequently used L2 VLS (see Table 10).

**Table 10:** Indicators of VLSs (N = 230).

Indicators						
Items	N	Minimum	Maximum	M	SD	Median
VLSM1	230	1	5	2.887	0.996	3
VLSM2	230	1	5	2.639	1.104	3
VLSM3	230	1	5	2.987	1.009	3
VLSM4	230	1	5	2.865	0.991	3
VLSM5	230	1	5	2.887	0.991	3
VLSM6	230	1	5	2.913	1.003	3
VLSM7	230	1	5	3.061	0.965	3
VLSM8	230	1	5	3.07	0.923	3
VLSM9	230	1	5	3.157	1.029	3
VLSM10	230	1	5	3.217	1.009	3
VLSM11	230	1	5	3.235	1.005	3
VLSM12	230	1	5	2.774	1.024	3
VLSM13	230	1	5	3.083	0.956	3
Average				2.98	1	
VLSC1	230	1	5	3.204	1.005	3
VLSC2	230	1	5	3.326	1.075	3
VLSC3	230	1	5	3.474	1.009	3
VLSC4	230	1	5	3.322	1.016	3
VLSC5	230	1	5	3.365	0.97	3
VLSC6	230	1	5	3.083	0.943	3
VLSC7	230	1	5	3.026	0.925	3
VLSC8	230	1	5	2.843	1.003	3
VLSC9	230	1	5	3.074	0.934	3
Average				3.191	0.967	
VLSE1	230	1	5	2.878	0.99	3
VLSE2	230	1	5	3	0.98	3
VLSE3	230	1	5	3.03	0.936	3
VLSE4	230	1	5	2.974	0.98	3
VLSE5	230	1	5	2.735	1.004	3
VLSE6	230	1	5	2.843	1.033	3
VLSE7	230	1	5	2.761	1.057	3
VLSE8	230	1	5	2.743	1.032	3
VLSE9	230	1	5	2.991	0.896	3
VLSE10	230	1	5	2.93	0.95	3
Average				2.889	0.986	
Overall M ± SD = 3.02 ± 0.984						

As for English learners with different genders (male/female), data showed that mean scores and SD of cognitive strategies were M ± SD = 2.79 ± 0.98, 3.12 ± 0.98, indicating that both male and female students preferred cognitive strategies. Cognitive strategies were also preferred by students from different grades and majors (see Table 11). As for students who studies in different high school, no matter from the urban or the rural areas, they all loved to apply cognitive strategies to study vocabularies in learning English as a second language, with M ± SD = 3.19 ± 0.98. From the data, it is concluded the sampled Chinese college students would like to apply cognitive strategies in learning vocabularies, which was consistent with the above-mentioned data in Table 10: M ± SD = 3.191 ± 0.967 (see Table 10).

**Table 11:** Preferred VLS of the English learners with different genders, grades, majors, and high school locations (N = 230).

Variables		VLS (M ± SD)			
		Memory	Cognitive	Meta-cognitive	Average
Genders	Male	2.72 ± 0.95	2.95 ± 1.02	2.71 ± 0.98	2.79 ± 0.98
	Female	3.09 ± 1.00	3.30 ± 0.95	2.97 ± 0.98	3.12 ± 0.98
	Average	2.91 ± 0.98	3.13 ± 0.99	2.84 ± 0.98	2.96 ± 0.98
Grades	Freshmen	3.02 ± 1.00	3.31 ± 0.93	2.90 ± 0.97	3.08 ± 0.97
	Sophomore	2.84 ± 0.97	2.91 ± 1.04	2.84 ± 1.01	2.86 ± 1.01
	Junior	2.78 ± 1.02	2.93 ± 1.05	2.78 ± 0.98	2.83 ± 1.02
	Senior	3.23 ± 0.84	4.00 ± 0.63	3.95 ± 0.60	3.73 ± 0.69
	Average	2.97 ± 0.96	3.29 ± 0.91	3.12 ± 0.89	3.13 ± 0.92
Majors	Humanities	3.02 ± 0.99	3.18 ± 0.96	2.97 ± 0.95	3.06 ± 0.97
	Science and engineering	2.94 ± 1.01	3.20 ± 1.00	2.82 ± 1.01	2.99 ± 1.01
	Average	2.91 ± 0.98	3.19 ± 0.98	2.90 ± 0.98	3.03 ± 0.99
Locations	Urban	3.09 ± 1.05	3.32 ± 1.02	2.99 ± 1.04	3.13 ± 1.04
	Rural	2.85 ± 0.93	3.05 ± 0.93	2.78 ± 0.92	2.89 ± 0.93
	Average	2.97 ± 0.99	3.19 ± 0.98	2.89 ± 0.98	3.01 ± 0.99

What should be figured out was that seniors in China had no traditional face-to-face English classes anymore, while they still faced pressure to study English, since some of them were preparing for the further studies to be undergraduates or to study abroad and some needed to catch the last chances to pass CET4. In this case, they may regulate their own vocabulary learning, monitoring their learning process, assessing themselves, adjusting themselves, and learning or practicing vocabulary with peers with relatively high intrinsic motivation.

**RQ3: What are the relationships between: (a) motivation and VLSs, (b) motivation and the VK breadth, (c) VLSs and the VK breadth, (d) motivation, VLSs, and the VK breadth? It is hypothesized that H1: EFL learners’ motivation has a direct positive effect on their use of VLSs, H2: EFL learners’ motivation has a direct positive effect on their VK breadth, H3: EFL learners’ VLSs positively and directly predict their VK breadth, and H4: If VLSs plays a mediating role, motivation will exert an indirect effect on VK breadth.**

**Fig 2:**
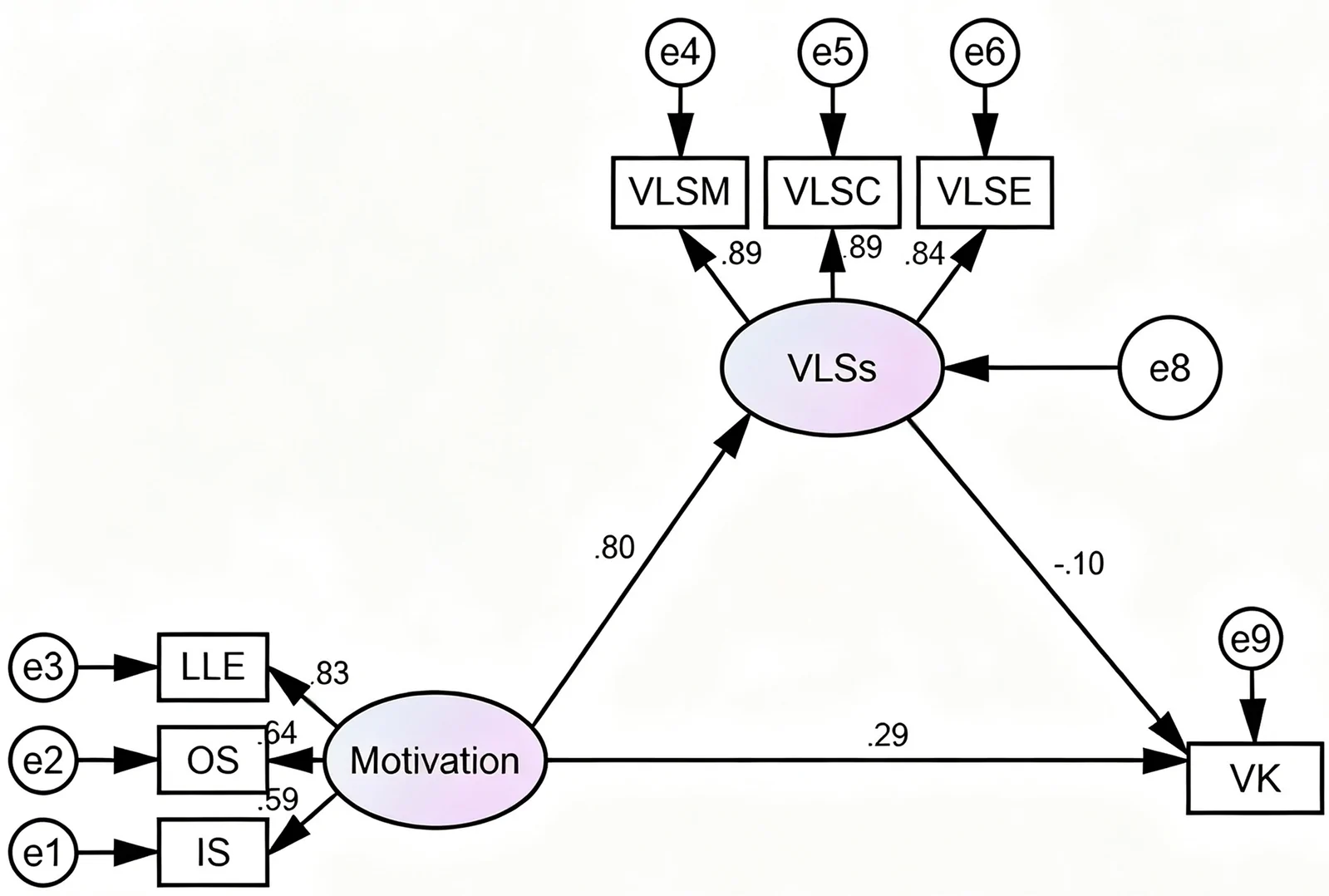
SEM of Motivation, VLSs, and VK.

A SEM of motivation, VLSs and VK was primarily conducted to test the model (see Fig 2). Data in Table 12 illustrated that CMIN = 36.737, DF = 12, CMIN/DF = 3.061, ranging from 3 to 5, meaning that The overall model fit was acceptable. The model demonstrated satisfactory performance on both absolute and incremental fit indices, suggesting a high goodness-of-fit, since GFI = 0.958, IFI = 0.970, NFI = 0.956, TLI = 0.947, CFI = 0.970, all higher than 0.90. RMSEA = 0.095 > 0.08. Hu and Bentler [82] proposed that that CFI/TLI/IFI values ≥ 0.90 indicate acceptable fit and ≥ 0.95 indicate excellent fit. Browne and Cudeck [83] suggested that RMSEA < 0.05 reflects close fit, 0.05–0.08 reasonable fit, and 0.08–0.10 mediocre but acceptable fit. While RMSEA = 0.095 slightly exceeds the commonly recommended threshold of 0.08, it remains within the “mediocre but acceptable” range [83], particularly for models with smaller sample sizes and moderate complexity. Some recent studies have also reported and interpreted RMSEA values of 0.095 as indicative of acceptable model fit when other fit indices meet recommended thresholds [84,85]. Overall, most fit indices of the model achieved excellent levels, whereas a few reached acceptable levels. These results suggest that the model demonstrates a good overall fit, reasonably captures the relationships among the variables, and satisfies the requirements for subsequent path analysis and hypothesis testing (see Table 12).

**Table 12:** SEM model fit results.

Fit Index	Acceptable Standard	Excellent Standard	Actual Value
CMIN	–	–	36.737
DF	–	–	12
CMIN/DF	3-5	<3	3.061
GFI	0.80-0.90	>0.90	0.958
RMSEA	0.08-0.10	<0.08	0.095
IFI	0.80-0.90	>0.90	0.970
NFI	0.80-0.90	>0.90	0.956
TLI(NNFI)	0.80-0.90	>0.90	0.947
CFI	0.08-0.90	>0.90	0.970

The standardized path coefficient from motivation to VLSs was 0.796, with a standard error of 0.089, C.R. = 10.006, p < 0.001, reaching a significant level, indicating that Motivation has a significant positive effect on the use of VLSs, which means the stronger the EFL learners’ learning motivation, the higher their level of VLS use. The standardized path coefficient from motivation to VK breadth was 0.294, with a standard error of 4.736, C.R. = 2.157, p = 0.031 < 0.05, also reaching a significant level, indicating that Motivation has a significant positive effect on VK breadth, suggesting that the stronger the learner’ learning motivation, the higher their level of VK breadth. The standardized path coefficient from VLSs to VK breadth was −0.097, with a standard error of 4.170, C.R. = −0.726, p = 0.468 > 0.05, failing to reach a significant level, indicating that the direct effect of VLSs on VK is not significant. Although this path coefficient is negative, it cannot be considered as evidence of a stable negative predictive effect of VLSs on VK breadth due to the failure to pass the significance test.

Overall, the direct path results of the model indicate that Motivation significantly and positively predicts both VLSs and VK, whereas the direct predictive effect of VLSs on VK is not significant (see Table 13).

**Table 13:** Results of the path model testing.

X	→	Y	Standardized Coefficients	S.E.	C.R.	P
Motivation	→	VLSs	0.796	0.089	10.006	<0.001
Motivation	→	VK	0.294	4.736	2.157	0.031
VLSs	→	VK	−0.097	4.17	−0.726	0.468

The mediation effect was tested by using AMOS 24.0 with 5,000 bootstrap resamples, applying the bias-corrected non-parametric percentile bootstrap method. As presented in Table 13, the estimated mediation effect was −2.692, with a 95% bootstrap confidence interval of [−13.901, 6.863] and a p = 0.554. As the confidence interval contained zero and the p-value exceeded 0.05, the mediation effect was not significant, which suggests that VLSs did not significantly mediate the relationship between motivation and VK. That is, while motivation significantly predicted both VLSs and VK, VLSs did not serve as a significant mediating mechanism in this relationship. Thus, the effect of motivation on VK was primarily direct rather than indirect via VLSs (see Table 14).

**Table 14:** Mediation analysis results.

Estimate	Lower	Upper	P
−2.692	−13.901	6.863	0.554

Data in Table 11 demonstrated that H1 and H2 were supported by the data, which means that motivation could positively influence the use of VLSs and the breadth of VK (H1:β = 0.796, p < 0.001; H2:β = 0.294, p = 0.031). Students who have a higher motivation may apply more VLSs in the English vocabulary learning, and they may also achieve a higher level of the VK breadth. To our surprise, H3 (β = −0.097, p = 0.468) and H4 (Estimate = −2.692, 95% CI [−13.901, 6.863], p = 0.554) were not supported by the data, which means that VLSs could not positively influence VK breadth, and that motivation will not indirectly predict the breadth of VK breadth if VLSs plays a mediating role. As for H3, it means that even with more VLSs, one could not necessarily acquire a higher level of the VK breadth. As for H4, VLSs have no mediation role between motivation and VK breadth (see Table 15).

**Table 15:** Results of the research hypothesis.

Hypothesis	Path/ Relationship	Expected Direction	Result	Supported
H1	Motivation → VLSs	Positive	β = 0.796, p < 0.001	Yes
H2	Motivation → VK (breadth)	Positive	β = 0.294, p = 0.031	Yes
H3	VLSs → VK (breadth)	Positive	β = -0.097, p = 0.468	No
H4	Motivation → VLSs → VK (breadth)	Indirect positive effect	Estimate = −2.692, 95% CI [−13.901, 6.863], p = 0.554	No

## 5. Discussion

The present study investigated the relationships among Chinese EFL learners’ motivation, VLSs, and VK breadth, with a specific focus on whether VLSs mediate the motivation-VK link. While several findings are consistent with prior research [10], the null relationship between VLSs and VK breadth—and the consequent absence of mediation—warrants careful theoretical consideration. Below, we discuss the primary findings, interpret their meaning in light of existing literature, and address potential explanations for unexpected results.

### 5.1. Direct effects of motivation on VLSs and VK breadth

Consistent with previous studies of Jang Ho Lee, Joung Joo Ahn, and Hansol Lee [18], Wen-Ta Tseng [86] and Jahangirzadeh, M., Ahangaran, F., & Pouyan, A [87]., motivation exerted a strong positive direct effect on VLSs use (β = 0.796, p < 0.001) and a moderate positive direct effect on VK breadth (β = 0.294, p = 0.031). These findings support the well-established premise that motivated learners are more likely to employ learning strategies and, independently, achieve better learning outcomes [9]. From a theoretical perspective, these results align with expectancy-value theory and self-determination theory: learners who value English learning and perceive themselves as competent tend to be both more strategic and more successful. Notably, the magnitude of the motivation-VLSs effect (β = 0.796) suggests a particularly strong association, indicating that for this cohort, motivation is a dominant driver of strategy use.

### 5.2. The null relationship between VLSs and VK breadth

The most unexpected finding is that VLSs did not significantly predict VK breadth (β = −0.097, p = 0.468), and consequently, motivation had no indirect effect through VLSs (Estimate = −2.692, 95% CI [−13.901, 6.863], p = 0.554). This result diverges from a substantial body of literature reporting positive associations between strategy use and vocabulary knowledge [4]. However, it is partially consistent with Ueno and Takeuchi [88], who found only a small correlation between VLSs and vocabulary size. Given that this null finding contradicts much of the existing literature, we consider several theoretically plausible explanations. Rather than treating the result as an isolated anomaly, we offer these as testable hypotheses for future research.

#### 5.2.1. Measurement artifacts: instrument difficulty.

A scientifically parsimonious explanation concerns the difficulty level of the VST. If the test was too difficult for the sampled students—who reported moderate, slightly below-average motivation—it may have failed to accurately capture the full range of students’ actual vocabulary knowledge, particularly at the lower end. Under such conditions, the statistical relationship between VLSs and VK breadth would be attenuated, potentially to non-significance. This measurement-based explanation is acknowledged as a primary limitation of the present study.

#### 5.2.2. Strategy quantity versus strategy quality.

Our VLSs questionnaire measured the frequency of strategy use, not the quality or effectiveness of that use. A learner may frequently employ cognitive strategies (e.g., repetition, word list memorization) but apply them ineffectively—for instance, using massed rather than spaced practice, or failing to engage in retrieval and elaboration. Conversely, another learner might use fewer strategies but deploy them with greater meta-cognitive regulation and deeper processing. The null finding suggests that the mere quantity of strategy use does not guarantee larger vocabulary size; what may matter more is how strategies are used. Future research should incorporate measures of strategy quality, such as depth of processing or adherence to evidence-based principles (e.g., spaced repetition, active recall).

#### 5.2.3. Aggregation of distinct strategy types.

Memory, cognitive, and meta-cognitive strategies were combined into a single latent variable. This aggregation may obscure differential—and potentially opposing—relationships with VK breadth. In the test-oriented Chinese context, cognitive strategies (e.g., rote repetition, word lists) might be positively associated with vocabulary size, as they directly target form-meaning mapping. Meta-cognitive strategies (e.g., planning, monitoring, evaluating), while beneficial for long-term development, might show a weaker or even negative cross-sectional correlation if learners only resort to them when they perceive difficulties. Aggregating these distinct strategy types into one latent variable could cancel out opposing effects, leading to a null overall relationship. Future research should model each strategy category as a separate mediator to examine whether differential indirect effects emerge.

#### 5.2.4. Direct pathway dominance.

The significant direct effect from motivation to VK breadth (β = 0.294, p = 0.031), combined with the null indirect effect via VLSs, suggests that motivation influences vocabulary knowledge through pathways other than self-reported strategy use. Potential pathways include increased exposure to English input (e.g., extensive reading, listening to media), greater persistence during challenging vocabulary tasks, or more effective use of classroom learning opportunities—none of which were captured by our VLSs questionnaire. This interpretation aligns with research showing that motivational constructs can directly predict language outcomes or be mediated by more proximal factors such as goal orientations or self-regulatory capacity [89], rather than by broad strategy inventories.

#### 5.2.5. Contextual factors specific to Chinese College EFL Learners.

Unlike senior secondary students who face intense pressure to perform on the National College Entrance Examination (Gaokao) [5], the sampled college students may experience reduced extrinsic pressure to expand their vocabulary. In the absence of high-stakes testing, the relationship between strategy use and vocabulary growth may weaken, as strategies are employed less consistently or effectively. Furthermore, the prevalent practice among Chinese college students of memorizing word lists from textbooks or using mobile applications—often without retrieval practice or contextualized review—may represent strategy use that is not particularly effective for long-term vocabulary retention. This contextual explanation does not negate the potential effectiveness of VLSs but rather highlights that the way strategies are implemented in this specific context may undermine their efficacy.

### 5.3. Comparison with Prior Literature

The direct effects of motivation on VLSs and VK breadth corroborate previous findings [18,86,87]. However, the null mediation effect diverges from studies that have reported significant indirect effects [18],. This discrepancy may be attributable to several factors. First, the present study’s sample (n = 230) was larger than that of Lee, Ahn, and Lee [18], (n = 185), potentially providing more stable estimates of null effects. Second, the educational contexts differ substantially: the present study examined Chinese university students, whereas Lee et al. [18],investigated Korean secondary school learners. Age, educational pressure, and language learning environments may moderate the mediating role of VLSs. Third, our use of a single aggregated VLSs latent variable, as noted above, may have masked mediation effects that would be detectable when analyzing each strategy type separately.

It is also worth noting that while VLSs did not mediate the motivation-VK relationship, motivation retained a significant direct effect. This pattern suggests that for this cohort, the primary pathway from motivation to vocabulary outcomes is not through self-reported strategy frequency. This finding aligns with a growing body of research questioning the assumed centrality of strategy mediation in L2 acquisition.

### 5.4. Pedagogical implications

Notwithstanding the null mediation finding, the results carry practical implications for English vocabulary instruction in Chinese higher education. First, given that motivation directly and positively predicts both VLSs use and VK breadth, instructors should prioritize motivation-enhancing activities. Blended learning, online vocabulary quizzes, inter-class competitions, and real-world communication tasks may stimulate learners’ intrinsic and extrinsic motivation. Second, strategy instruction should not be abandoned but re-conceptualized. Rather than simply encouraging more frequent strategy use, educators should focus on teaching effective strategy application—for example, spaced repetition, active recall, elaboration, and contextualized learning. The null finding suggests that merely using strategies more often does not guarantee better vocabulary outcomes.

Third, cognitive strategies emerged as the most preferred category among the sampled students (M = 3.191, SD = 0.967). While this preference may reflect the test-oriented learning culture, instructors could build upon this tendency by introducing more effective variants of cognitive strategies (e.g., keyword method, word mapping) while gradually introducing metacognitive strategies (e.g., self-monitoring, progress evaluation) to foster autonomous learning.

### 5.5. Limitations and Future Directions

Several limitations of the present study should be acknowledged. First, sample generalisability and grade distribution. The 230 validated responses were obtained from a university in Wuhan, central China, which may not represent other Chinese universities or other educational systems. Moreover, the grade distribution was substantially uneven: freshmen constituted 67.39% of the sample, sophomores 23.48%, juniors 8.26%, and seniors only 0.87% (two participants). This imbalance, particularly the under-representation of senior students, limits the generalisability of findings across grade levels and precludes robust subgroup comparisons. Second, cross-sectional design. The data are cross-sectional, which precludes causal inference regarding the direction of relationships. Longitudinal designs are needed to establish the temporal ordering of motivation, VLSs use, and vocabulary development. Third, common method bias. All measures were either self-reported or performance-based but collected within a single questionnaire session, which may inflate correlations among self-reported constructs. Future research should consider collecting predictor and criterion variables from different sources or at different time points. Fourth, aggregated strategy measure. Combining memory, cognitive, and metacognitive strategies into a single latent variable may obscure differential effects. As discussed in Section 5.2.3, future research should test separate mediation models for each strategy type to determine whether they operate through different mechanisms. Fifth, motivational subcomponents. The use of a composite motivation score masks the distinct roles of ideal L2 self, ought-to L2 self, and L2 learning experience. Future research should examine whether specific motivational subcomponents exert different direct or indirect effects on vocabulary outcomes. Sixth, vocabulary depth. The present study measured only vocabulary breadth. Vocabulary knowledge is multidimensional, encompassing both breadth (size) and depth (quality of knowledge). Future research should include measures of vocabulary depth to examine whether VLSs mediate the relationship between motivation and depth of knowledge. Seventh, exclusion of social and affective strategies. Social and affective VLSs, while acknowledged as important in the literature [4], were excluded from the questionnaire to maintain model parsimony and avoid excessive questionnaire length. This exclusion constitutes a limitation, and future research should examine whether these two strategy types play different mediating roles in the relationship between motivation and vocabulary knowledge.

## 6. Conclusions

This study examined the relationships among Chinese EFL learners’ motivation, VLSs, and VK breadth using structural equation modeling. While motivation exerted strong direct effects on both VLSs use and VK breadth, VLSs did not mediate the motivation-VK relationship. The null finding is interpreted in light of five potential explanations: measurement artifacts (instrument difficulty), the distinction between strategy quantity and quality, aggregation of distinct strategy types, direct pathway dominance, and contextual factors specific to Chinese college EFL learners. These findings caution against assuming a universal mediating role of VLSs and highlight the need for future research to examine strategy quality, separate strategy categories, and contextual moderators. Pedagogically, enhancing motivation and teaching effective strategy application—rather than simply encouraging frequent strategy use—may be more productive pathways to vocabulary development.

## Supporting information

S1 File Questionnaire and report-English-language version. (XLSX)

S2 File Vocabulary-Size-Test-information-and-specifications. (PDF)
