# Supplementary material for: Relationship among motivation, vocabulary learning strategies and the breadth of vocabulary knowledge: A structural equation modeling study on second language learning
Source: PLoS One. 2026 Sep 22;21(9):e0356062. doi: 10.1371/journal.pone.0356062 (PMC13596826; doi:10.1371/journal.pone.0356062)
Supplement: Supplementary file 1 [file pone.0356062.s002.pdf]

The Vocabulary Size Test

Paul Nation 23 October 2012

Available versions

There is a 14,000 version containing 140 multiple-choice items, with 10 items from each 1000 word family level. A learner's total score needs to be multiplied by 100 to get their total receptive vocabulary size.

There are two more recent parallel 20,000 versions each containing 100 multiple choice items. A learner's total score needs to be multiplied by 200 to get their total receptive vocabulary size. The two forms have been tested for their equivalence.

Permission is not required to use these tests in research, although acknowledgement in any thesis or publication is appreciated. The reference for the 14,000 level test is Nation, I.S.P. & Beglar, D. (2007) A vocabulary size test. *The Language Teacher*, *31*(7), 9-13. (Check Publications on Paul Nation's web site for current information on publications).

Goals and construct

The Vocabulary Size Test is designed to measure both first language and second language learners' written receptive vocabulary size in English.

The test measures knowledge of written word form, the form-meaning connection, and to a smaller degree concept knowledge. The test measures largely decontextualised knowledge of the word although the tested word appears in a single non-defining context in the test.

Users of the test need to be clear what the test is measuring and not measuring. It is measuring written receptive vocabulary knowledge, that is the vocabulary knowledge required for reading. It is not measuring listening vocabulary size, or the vocabulary knowledge needed for speaking and writing. It is also not a measure of reading skill, because although vocabulary size is a critical factor in reading, it is only a part of the reading skill. Because the test is a measure of *receptive* vocabulary size, a test-taker's score provides little indication of how well these words could be used in speaking and writing.

Using Read and Chapelle's (2001) framework, the Vocabulary Size Test is a discrete, selective, relatively context-independent vocabulary test presented in a multiple-choice format. The test is available in monolingual and bilingual versions testing up to the 20th 1000 word level. Test-takers are required to select the best definition or translation of each word from four choices. The test is available in hard copy and computerised formats.

Inferences: Although the tested words are presented in simple non-defining contexts, it is essentially following a trait-definition of vocabulary which means that vocabulary knowledge is tested independently from contexts of use. At the item level, the test measures receptive knowledge of a written word form. At the test level it provides an estimate of total vocabulary size where vocabulary knowledge is considered as

including only single words (not multiword units) and vocabulary size does not include proper nouns, transparent compounds, marginal words like *um, er, gee gosh,* and abbreviations. It does not measure the ability to distinguish homonyms and homographs.

Uses: For instructional purposes the results can be used to guide syllabus design, extensive reading, and vocabulary instruction. For research purposes, it can be used a measure of total receptive written vocabulary size for both native and non-native speakers.

Impacts: If it is used as intended, it is a relatively low stakes test for learners. One consequence may be that it substantially underestimates the vocabulary size of learners who are not motivated to perform to the best of their ability, especially if they are judged to be low achievers within their education system. This could result in faulty instructional decisions being made about their vocabulary learning needs, and thus the test may need to administered orally to such students on a one-to-one basis. . More generally, the discrete, context-independent nature of the test format may encourage the study of isolated words.

Washback

The Vocabulary Size Test is primarily a test of decontextualised receptive knowledge of written vocabulary. Such a test could encourage the decontextualised learning of vocabulary. Such learning is to be encouraged, because (1) decontextualised learning using word cards or flash card programs is highly efficient (Nation, 2001: 297-299, and (2) such learning results in both explicit and implicit knowledge (Elgort, 2011).

Specifications for making the test

Sampling the words for the items

The items in the test need to represent the various frequency levels of the language without a bias towards any particular frequency levels. The frequency levels are based on word families occurring in the British National Corpus according to Bauer and Nation's (1993) levels up to Level 6.

Because the goal of the test is to measure total vocabulary size, the test should measure frequency levels beyond the test-takers' likely vocabulary size. For this reason, only a small number of items can be sampled from each vocabulary level. The test uses frequency levels based on the British National Corpus word family lists for the sampling, but the tests do not reliably measure how well each level is known, because there are not enough items at each level. We expect scores to decrease for the levels. The total score for the test is what matters.

Words that are loanwords or cognates in the learner’s first language are not removed from the test. Removing the items would distort the measurement of vocabulary size, because loanwords and cognates are a legitimate part of a learner’s second language vocabulary size. The Vocabulary Size Test thus measures words known rather than words learnt.

Making the stem

The test uses a stem plus a 4 choice multiple-choice format. The item stem consists of the word followed by a very simple non-defining sentence containing the word. The non-defining sentence has the roles of (1) indicating the part of speech of the word, (2) limiting the meaning of the word where words may have a homograph or very different senses, and (3) slightly cueing the meaning by presenting an example of use. The words represented by the distractors should fit sensibly within the stem. The vocabulary of the stem (with the exception of the tested word) is within the first 500 words of English.

Writing the choices

The distractors are the same part of speech as the correct answer, and in most cases the distractors are the meanings of words from around the same 1000 word frequency level as the correct answer.

59. emir: We saw the <emir>.

a	bird with two long curved tail feathers	[peacock]
b	woman who cares for other people's children in eastern countries	[amah}
c	Middle Eastern chief with power in his own land	[emir]
d	house made from blocks of ice	[igloo]

Non-meaning clues such as the length of the choice, and general versus specific choices have been avoided and have been checked in piloting.

The occurrence of the correct answers is roughly spread evenly across the four choices of *a, b, c, d.*

As much as possible, the test is a measure only of vocabulary knowledge and not of vocabulary in use. Because of its focus on vocabulary, sitting the test should require very little knowledge beyond vocabulary knowledge and reading skill. For this reason, the choices are written in much easier language than the tested word. For the first and second 1000 word levels, only words from the first 1000 of West's (1953) General Service List were used. As far as possible, the words in the definitions were of higher frequency than the item being defined, but for the highest frequency items, this was not always possible, e.g., there was no possibility for defining *time* except with words of lower frequency (e.g. *hours*). For words from the 3000 word level upwards, the defining words were drawn from the first 2000 of West's General Service List.

If bilingual test items are acceptable, the test should be bilingual. Here is an example of a monolingual item and a bilingual item.

1. soldier: He is a **soldier**.
   a. person in a business
   b. student
   c. person who uses metal
   d. person in the army

1. soldier: He is a **soldier**.
   a. 商人
   b. 学生
   c. 金属工艺制造者
   d. 士兵

Elgort (in press) found that sitting the bilingual version of the test resulted in scores around 10% higher. The reasons for the higher scores are likely to be because

translations avoid the difficult grammar of English definitions and they are immediately comprehensible to the test takers.

Using first language translations does not mean translating definitions into the first language. It means providing a single first language word or phrase for each choice. That is, the choices are first language synonyms of a second language word.

The test items do not require full knowledge of each word, but allow learners to use partial knowledge. Partial knowledge is allowed for by having distractors that do not share core elements of meaning with the correct answer. So, the item testing *azalea* does not require the learners to distinguish between various types of plants, but simply to know that an azalea is a plant.

azalea: This **azalea** is very pretty.
a. small tree with many flowers growing in groups
b. light material made from natural threads
c. long piece of material worn by women in India
d. sea shell shaped like a fan

The test does not use an *I don't know option*, because such an option discourages informed guessing. The learners should make informed guesses, because these guesses are likely to draw on sub-conscious knowledge.

The test is a measure of written receptive vocabulary knowledge, that is, the kind of knowledge that is needed for reading. When reading, vocabulary knowledge is supported by background knowledge, reading skill, and textual context. Full knowledge of the meaning of the word is not required for reading, although the better the words are known, the easier the reading will be.

In addition, a major use of the test will be to guide learners in their vocabulary learning. If learners already have a partial but usable knowledge of some words, they should not be studying these words, but should move on to unknown vocabulary.

The order of the items in the test

Learners need to sit all of the items in the test because for various reasons learners are likely to get some items correct which are outside their typical level of vocabulary knowledge. These reasons include the presence of loanwords, and the presence of words related to hobbies, academic study, or specialist interests. Nguyen and Nation (2011) found that even lower proficiency learners improved their scores by sitting the lower frequency sections of the test.

The items in the test are usually arranged in frequency order. The frequency order may lead learners to give up during the later levels, so it is probably better to mix the levels, with higher frequency words appearing through the whole test. Such an order is more likely to maintain engagement with the test.

Piloting

Versions of the tests have been piloted in several ways.

1 Getting applied linguists who are native speakers of English to individually read and critique the test.
2 Replacing the target word with the nonsense word and getting a test-wise native speaker to try to choose the correct answer. This checked if the choices themselves were indicating the correct answer.
3 Running the tests through the Range program to check the frequency levels of words used in the contexts and choices.
4 A Rasch-based analysis was conducted using just under 200 students in Japan (Beglar, 2010).

Using the Vocabulary Size Test

Administration of the test

The test is a measure of knowledge not fluency, and so enough time should be given to complete the test and allow learners to ponder over each item. It typically takes around 40 minutes to sit the 140 item test, and around 30 minutes for the 100 item tests.

The validity of any test depends strongly on how seriously learners sit the test. If they simply skip through it while playing with their cell phones, the results will be meaningless. For some learners, it may be necessary to administer the test on a one-to-one basis. This type of administration can include providing help by pronouncing unfamiliar words for the test-taker, encouraging them, and giving them feedback on already completed items. For some learners, a one-to-one administration of the test can double the score that they got on a group-administered test.

The test is suitable for computer-based delivery and scoring.

Test equivalence

Versions A and B of Vocabulary Size Test are parallel forms. It is relatively straightforward to make parallel forms of the Vocabulary Size Test because it is largely a unidimensional measure (Beglar, 2010) and the specifications described in this document are easy enough to follow. The various forms of the test have been trialled with 46 people who sat all forms of the test. The means and standard deviations of versions A and B were close to each other and not significantly different (Version A mean 81.37, sd 16.662; Version B mean 83.20, sd 13.982). This means that versions A and B can be used as if they were the same test.

Scoring the test

When scoring the test, the 1000 frequency levels can be ignored. The levels are there simply to make sure that the test is not biased to any particular level.

A learner’s total score on the 140 item test needs to be multiplied by 100 to find the learner's total vocabulary size. So, a score of 35 out of 140 means that the learner's vocabulary size is 3,500 word families. On the 100 item versions measuring up to the 20th 1000 word family level, there are five words for each 1000 word family level, so the total score needs to be multiplied by 200.

Correction for guessing

There should not be a correction to guessing. This is because a correction would distort the measurement of vocabulary size, because each tested word represents 100 or 200 words. We need to assume that learners are not making wild guesses. The interpretation of the final scores however needs to be done with the understanding that, because the test is a partially sensitive test and because there is no correction for guessing, the vocabulary size score is a slightly generous estimate of vocabulary size.

Interpreting the scores

To work out what the score means in terms of language use, we need to look at the vocabulary size needed to gain a text coverage of 98% in various kinds of texts.

Table 1: Vocabulary sizes needed to get 98% coverage (including proper nouns) of various kinds of texts (Nation, 2006)

Texts	*98% coverage*	*Proper nouns*
Novels	9,000 word families	1-2%
Newspapers	8,000 word families	5-6%
Children's movies	6,000 word families	1.5%
Spoken English	7,000 word families	1.3%

The goal of around 8,000 word families is an important one for learners who wish to deal with a range of unsimplified spoken and written texts. It is thus helpful to know how close learners are to this critical goal.

Initial studies using the test indicate that undergraduate non-native speakers of non-European backgrounds successfully coping with study at an English speaking university have a vocabulary size around 5,000-6,000 word families. Non-native speaking PhD students have around a 9,000 word vocabulary.

To work out what learners should be doing to increase their vocabulary size, we need to relate the vocabulary size score to the three main frequency levels of high-frequency, mid-frequency, and low-frequency words.

**Level**	**1000 word family lists**	**Learning procedures**
High frequency	1000-2000	Reading graded readers<br>Deliberate teaching and learning
Mid-frequency	3000-9000	Reading mid-frequency readers<br>Deliberate learning
Low frequency	10,000 on	Wide reading<br>Specialised study of a subject area

The Vocabulary Size Test can be used to test both native speakers or non-native speakers. The general rule of thumb for predicting the vocabulary size of young native speakers is to take two or three years away from the age of the native speaker and multiplied this figure by 1000. So, an average 13 year old native speaker knows

between 10,000 and 11,000 word families receptively (13-2 = 11 x1000 = 11,000). At any age level however, there may be a wide range of vocabulary sizes.

Studies using the test

A good vocabulary test has the following features and Beglar's (2010) examination of the 140 item Vocabulary Size Test showed that it does have these features.

1 It can be used with learners with a very wide range of proficiency levels.
2 It measures what it is supposed to measure and does not measure other things. Beglar found that the test was very clearly measuring a single factor (presumably written receptive vocabulary knowledge) and other factors played a very minor role in performance on the text.
3 It behaves in ways that we would expect it to behave, distinguishing between learners of different proficiency levels, having a range of item difficulties related to the frequency level of the tested words, and clearly distinguishing several different levels of vocabulary knowledge so that learners' vocabulary growth over time could be measured.
4 It performs consistently and reliably, even though circumstances change. In Beglar's trialling of the test, these changes included comparing the performance of male subjects with female subjects, comparing 70 item versions of the test with the 140 item version, and comparing learners of various proficiency levels. Rasch reliability measures were around .96.
5 It is easy to score and interpret the scores.
6 The items in the test are clear and unambiguous.
7 It can be administered in efficient ways with learners sitting only five words per 1000 word level.

The 140 item test works very well because it covers a very wide range of frequency levels, it includes a large number of items (even half of this number would work well), the items have been very carefully designed and made, and the test is designed to measure just one kind of vocabulary knowledge.

Nguyen and Nation (2011) showed that it is important to sit all levels of the test because for various reasons some words at the lower frequency levels will be known. This may be because they are loan words or cognates, because they relate to learners hobbies and interests, because they are technical words in fields the learners are familiar with, or because the learners just happened to meet them.

An as yet unpublished study shows that when there is an "I don't know" choice

1 the test takes less time. This shows that learners are carefully considering options and are not making wild guesses when there is no "I don't know" option.
2 learners' scores are lower. This shows that discouraging guessing means learners cannot get credit for items where they have some partial knowledge of the word.
3 their receptive multiple-choice scores are closer to their receptive recall scores. This shows that discouraging guessing turns a sensitive test into a tougher test.

4 a penalty for making an incorrect answer instead of choosing "I don't know" increases the choice of the "I don't know" option by around a third. This shows that if you want to use a multiple-choice test as a tough test of knowledge rather than as a sensitive test of knowledge giving credit for partial knowledge, then adding an "I don't know option" should be accompanied by a penalty that the learners know about.

An important reason for using a multiple-choice format for a vocabulary test is to allow learners to draw on partial knowledge when they answer the test. That is, the choice of that particular format can be because the goal of the test maker is to give as much credit as possible for what learners know even if this knowledge is incomplete. If the test is designed for this purpose, then test-takers should be encouraged to use their intuition and make informed or intuitive guesses.

Sources of words for the tests

The British National Corpus word family lists

The words to be tested are sampled from the British National Corpus word family lists which now go up to the 25th 1000. The British National Corpus word lists (more recently the BNC/COCA wordlists) are lists of word families developed for two main purposes.

1 The lists are designed to be used in the Range and AntWordProfiler programs to analyse the vocabulary load of texts.
2 The lists are designed to be as complete as possible lists of the high-frequency (1000-2000), mid-frequency (3000-9000) words. They also cover 16,000 low-frequency words which include the more common low-frequency words.

For information on the British National Corpus wordlists see Paul Nation’s web site.

Word families

The unit of counting in the British National Corpus frequency lists, and therefore the unit of counting in the test, is the word family. The word family was chosen, rather than the word type or lemma, firstly because research has shown that word families are psychologically real (Nagy, Anderson, Schommer, Scott & Stallman, 1989; Bertram, Laine & Virkkala, 2000). Secondly, when reading, knowing one member of the family and having control of the most common and regular word-building processes makes it possible to work out the meaning of previously unmet members of the family. Here are some examples of low-frequency family members of high-frequency word families – *burningly, helplessness, snappishly, curiouser*.

The unit of counting makes a substantial difference to the number of words in the language. For example, the first 1000 word families contain 6,857 word types, which means each word family at this level has an average of just under seven words per family. The average number of types per family decreases as you move down the frequency levels. When measuring vocabulary knowledge for productive purposes, the word type or the lemma is a more sensible unit of counting. If the lemma was the unit of counting for receptive purposes, the following items would be counted as

different words – *walk* (verb), *walk* (noun), *walking* (adjective), *walker*. These are all members of the same word family.

Links

You can find the 14,000 test at these sites. Bilingual versions are also available.
http://www.victoria.ac.nz/lals/staff/paul-nation.aspx
http://jalt-publications.org/tlt/resources/2007/0707a.pdf

Online versions of the test are available at
http://my.vocabularysize.com
http://www.lextutor.ca/

You can find the 20,000 versions A and B on these sites.

http://www.victoria.ac.nz/lals/staff/paul-nation.aspx

References

Bauer, L., & Nation, I. S. P. (1993). Word families. *International Journal of Lexicography*, *6*(4), 253-279.

Beglar, D. (2010) A Rasch-based validation of the Vocabulary Size Test. *Language Testing*, *27*(1), 101-118.

Bertram, R., Laine, M., & Virkkala, M. (2000). The role of derivational morphology in vocabulary acquisition: Get by with a little help from my morpheme friends. *Scandinavian Journal of Psychology, 41*(4), 287-296.

Elgort, I. (2011). Deliberate learning and vocabulary acquisition in a second language. *Language Learning, 61*(2), 367-413.

Nagy, W. E., Anderson, R., Schommer, M., Scott , J. A., & Stallman, A. (1989). Morphological families in the internal lexicon. *Reading Research Quarterly, 24*(3), 263-282.

Nation, I. S. P., (2006). How large a vocabulary is needed for reading and listening? *Canadian Modern Language Review, 63*(1), 59-82.

Nation, I.S.P. and Beglar, D. (2007) A vocabulary size test. *The Language Teacher, 31*(7), 9-13.

Nguyen, L. T. C., & Nation, I. S. P. (2011). A bilingual vocabulary size test of English for Vietnamese learners. *RELC Journal*, *42*(1), 86-99.

Read, J., & Chapelle, C. (2001). A framework for second language vocabulary assessment. *Language Testing*, *18*(1), 3-32.

Schmitt, N., Schmitt, D., & Clapham, C., (2001). Developing and exploring the behaviour of two new versions of the Vocabulary Levels Test. *Language Testing*, *18*(1), 55-88.

West, M. (1953). *A General Service List of English Words*. London: Longman, Green & Co.
